# Exploring whether ChatGPT-4 with image analysis capabilities can diagnose osteosarcoma from X-ray images

**DOI:** 10.1186/s40164-024-00537-z

**Published:** 2024-07-27

**Authors:** Yi Ren, Yusheng Guo, Qingliu He, Zhixuan Cheng, Qiming Huang, Lian Yang

**Affiliations:** 1 Department of Radiology, Union Hospital, Tongji Medical College, Huazhong University of Science and Technology, No.1277 Jiefang Avenue, Wuhan, 430022 China; 2 Hubei Key Laboratory of Molecular Imaging, Wuhan, 430022 China; 3 Department of Urology, The Second Affiliated Hospital of Fujian Medical University, Quanzhou, 362000 China; 4 Department of Urology, Union Hospital, Tongji Medical College, Huazhong University of Science and Technology, Wuhan, 430022 China; 5 Department of Invasive Technology, The Second Affiliated Hospital of Fujian Medical University, Quanzhou, 362000 China

**Keywords:** ChatGPT-4, X-ray image, Osteosarcoma, Diagnosis

## Abstract

The generation of radiological results from image data represents a pivotal aspect of medical image analysis. The latest iteration of ChatGPT-4, a large multimodal model that integrates both text and image inputs, including dermatoscopy images, histology images, and X-ray images, has attracted considerable attention in the field of radiology. To further investigate the performance of ChatGPT-4 in medical image recognition, we examined the ability of ChatGPT-4 to recognize credible osteosarcoma X-ray images. The results demonstrated that ChatGPT-4 can more accurately diagnose bone with or without significant space-occupying lesions but has a limited ability to differentiate between malignant lesions in bone compared to adjacent normal tissue. Thus far, the current capabilities of ChatGPT-4 are insufficient to make a reliable imaging diagnosis of osteosarcoma. Therefore, users should be aware of the limitations of this technology.

**Supplementary Information:**

The online version contains supplementary material available at 10.1186/s40164-024-00537-z.

## To the editor,

The generation of radiological results from image data is essential for medical image analysis. The most recent version of ChatGPT-4(Generative Pre-Training Transformer), a large multimodal model capable of integrating text and image inputs such as dermatoscopic [1], pathology [2], and X-ray images simultaneously [3, 4], is of significant interest to the field of radiology. In order to assess the performance of ChatGPT-4 in medical imaging image recognition, we conducted a study to evaluate its ability to accurately identify osteosarcoma X-ray images from real-world datasets.

**Fig. 1:**
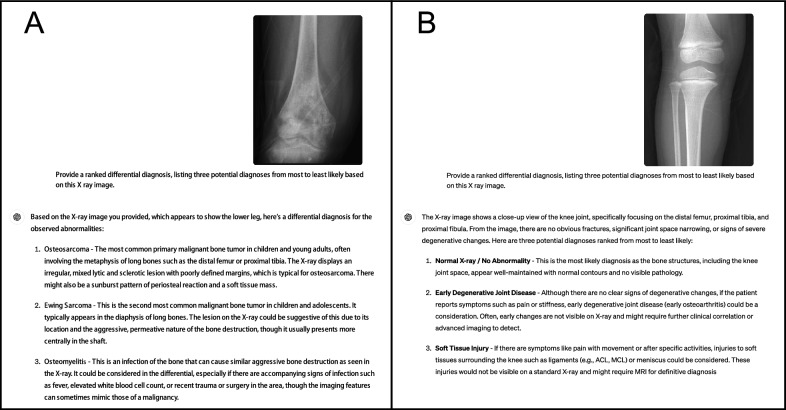
Example of dialogue with ChatGPT-4 V providing a brief description of the X-ray image picture and a differential diagnosis of the bone X-ray image picture. **A** Lower limb osteosarcoma. **B** Normal lower limb bone

We conducted a random selection of 40 cases each of lower limb osteosarcoma and normal lower limb bone X-ray images from the Picture Archiving and Communication System (PACS), ensuring that the images displayed typical characteristics of the condition and were accompanied by a pathological diagnosis of osteosarcoma. Following this, we obtained magnified representative images of lower limb osteosarcomas alongside images of normal lower limb bones. Specifically, to suggest the relationship between ChatGPT-4 lesions and adjacent anatomical structures, each of the osteosarcoma images contained portions of normal bone (Fig. 1A). However, the lesion sites remained more prominent in the images, as evidenced by the results of ChatGTP-4 identification of the presence or absence of occupying lesions (Table 1). In order to align with the osteosarcoma images, the normal lower limb bone images were also partially enlarged to include joints and half of the long bones (Fig. 1B). All chosen images underwent validation by a senior radiologist (L.Y.) before being inputted into ChatGPT-4 ('December 2023 version') on the dates of May 11th to May 12th, 2024. The model was tasked with generating a ranked differential diagnosis, presenting three potential diagnoses in order of likelihood (Fig. 1). To avoid affecting the performance of ChatGPT-4, we did not give it any prior prompts, and normal bone images and osteosarcoma images were entered randomly into ChatGPT-4 [5]. X-ray images of osteosarcoma and normal bone were randomly inputted for the primary (Top-1) diagnosis and the priority diagnosis derived from the first three potential diagnoses. The target outcome, ranging from coarse to fine, includes identifying between occupying and non-occupying lesions, as well as between malignant and non-malignant lesions, osteosarcoma and non-osteosarcoma. Sensitivity, specificity, and overall diagnostic accuracy were calculated using the stats and epiR packages in R (version 4.2.1). The efficacy of ChatGPT-4 in detecting the presence or absence of occupying lesions, malignant lesions, and lesions indicative of osteosarcoma on bone X-ray images was evaluated.

**Table 1:** Sensitivity, specificity, and diagnostic accuracy of ChatGPT-4 in diagnosing osteosarcoma

Group	Sensitivity (95% CI)	Specificity (95% CI)	Accuracy (95% CI)
Occupied vs non-occupied			
Top 1	55.0% (39.8–69.3)	100.0% (91.2–100.0)	77.5% (67.2–85.3)
Priority	65.0% (49.5–77.9)	100.0% (91.2–100.0)	82.5% (72.7–89.3)
Malignant vs non-malignant			
Top 1	37.5% (24.2–53.0)	100.0% (91.2–100.0)	68.8% (57.9–77.8)
Priority	52.5% (37.5–67.1)	100.0% (91.2–100.0)	76.3% (65.9–84.2)
Correct diagnosis			
Top 1	20.0% (10.5–34.8)	100.0% (91.2–100.0)	60.0% (49.0–70.0)
Priority	35.0% (22.1–50.5)	100.0% (91.2–100.0)	67.5% (56.6–76.8)

According to the findings of the ChatGPT-4 analysis on osteosarcoma and normal bone production, various types of bone-occupying lesions were categorized into the occupied group, with primary and secondary malignant bone tumors, as well as bone tumors exhibiting malignant tendencies, being classified as malignant. Conversely, normal bone, deformities, and other non-occupying bone diseases were categorized into the non-occupying group. The results showed that ChatGPT-4 was effective in diagnosing bone with or without occupying lesions, achieving accuracies of 0.825 and 0.775 for priority and Top-1 diagnosis, respectively (Table 1). The sensitivities were 0.650 and 0.550, respectively, and the specificities were both 1; secondly, ChatGPT-4 exhibited higher accuracy in distinguishing between malignant and non-malignant bone conditions during priority diagnosis, achieving an accuracy of 0.763, a sensitivity of 0.525, and a specificity of 1. However, its performance in the Top-1 diagnosis was comparatively lower, with an accuracy of 0.688, a sensitivity of 0.375, and a specificity of 1. Moreover, ChatGPT-4 demonstrated limited proficiency in identifying osteosarcoma, as evidenced by priority diagnosis and Top-1 diagnosis accuracies of 0.675 and 0.600, respectively. The sensitivities for osteosarcoma detection were 0.350 and 0.200, while the specificities remained consistently high at 1. It is noteworthy that the sensitivity is low and the specificity is high in all the above results, suggesting that ChatGPT-4 is very accurate in detecting images without bone lesions. In addition, the most frequent misdiagnoses were giant cell tumour of bone (Top1: 5, top2: 1, top3: 1) and bone metastases (Top1: 5, top2: 1, top3: 2) (Supplementary Material).

Our assessment is constrained by several limitations, including a relatively small sample size, a lack of patient background information such as age and gender compared to typical clinical scenarios, and the absence of bilateral contrast images. Additionally, factors such as tumor size, shape, location, border definition, type of periosteal reaction, and pattern of bone destruction were not considered in the analysis. Our future research aims to increase the sample size and conduct replicated experiments to mitigate the impact of randomness, because ChatGPT-4 may provide varying responses to identical queries. Additionally, we intend to investigate the various factors that influence the diagnostic accuracy of ChatGPT-4 in detecting osteosarcoma. Despite being an exploratory study, our findings offer valuable insights into the potential application of ChatGPT-4 in medical imaging diagnosis. In conclusion, while ChatGPT-4 shows potential for enhancing various aspects of medical practice and can diagnose bone conditions with or without significant space-occupying lesions, these limitations should be taken into account when interpreting the results.

### Supplementary Information

Supplementary Material 1.

## Data Availability

Raw data already included in Supplementary Material 1.

## Abbreviation

PACS: Picture archiving and communication system
